# Interleukin-1α enhances the aggressive behavior of pancreatic cancer cells by regulating the α_6_β_1_-integrin and urokinase plasminogen activator receptor expression

**DOI:** 10.1186/1471-2121-7-8

**Published:** 2006-02-20

**Authors:** Hirozumi Sawai, Yuji Okada, Hitoshi Funahashi, Yoichi Matsuo, Hiroki Takahashi, Hiromitsu Takeyama, Tadao Manabe

**Affiliations:** 1 Department of Gastroenterological Surgery, Nagoya City University Graduate School of Medical Sciences, Nagoya 4678601, Japan

## Abstract

**Background:**

In human pancreatic cancer progression, the α_6_β_1_-integrin is expressed on cancer cell surface during invasion and metastasis formation. In this study, we investigated whether interleukin (IL)-1α induces the alterations of integrin subunits and urokinase plasminogen activator/urokinase plasminogen activator receptor (uPA/uPAR) expression in pancreatic cancer cells. We hypothesize that the alterations of integrin subunits and uPA/uPAR expression make an important role in signaling pathways responsible for biological behavior of pancreatic cancer cells.

**Results:**

IL-1α upregulated the expression of α_6 _and β_1 _integrins without any alterations of α_5 _and α_v _integrins expression. IL-1α also induced enhancement in the expression of uPA/uPAR in pancreatic cancer cells. IL-1α enhanced the proliferation, adhesion, and migration in pancreatic cancer cells, and IL-1α-induced alterations of uPA/uPAR expression correlated with the increased the migration of pancreatic cancer cells. Upregulation of α_6 _integrin subunit and uPA/uPAR correlated with the activation of Ras and downstream extracellular signal-regulated kinase (ERK) pathways. IL-1α-induced activation of Ras and downstream ERK can be inhibited by using inhibitory antibodies against α_6 _and β_1 _integrin and uPAR, consistent with the inhibition of proliferation, adhesion and migration of pancreatic cancer cells. Immunohistochemical analysis demonstrated a significant association between strong expressions of α_6 _integrin with uPAR in pancreatic cancer specimens. Furthermore, the strong expression of α_6 _integrin and uPAR was found to be independent prognosticator in pancreatic cancer patients.

**Conclusion:**

Based on these findings, we conclude that IL-1α can induce selective upregulation of α_6_β_1_-integrin and uPA/uPAR in pancreatic cancer cells and these changes may modulate the aggressive functions of pancreatic cancer.

## Background

Pancreatic cancer is one of the most aggressive common tumors, the five-year survival rate being less than 20% despite surgery and/or chemotherapy [1]. This very poor prognosis is mainly due to the propensity of this tumor to invade the adjacent structures and metastasize to distant organs early in the course of disease. Despite intensive efforts to improve therapy for this advanced disease, treatment remains unsatisfactory and most patients die within months as a result of rapid local spread of the tumor or metastatic dissemination. The biological characteristics underlying the aggressive behavior of these tumors are incompletely understood.

Integrins are dimeric proteins composed of noncovalently associated α and β subunits and are divided into subgroups according to their preference for binding to extracellular matrix (ECM) proteins or cell surface molecules [2-4]. These adhesion molecules play principal roles in various aspects of tumor biology. Increased expression of laminin binding integrins or decreased expression of fibronectin binding integrins has been correlated with aggressive growth and metastatic capacity of several tumors [5-8]. We previously reported that the enhancement of α_6_β_1_-integrin expression by interleukin (IL)-1α acting through IL receptor type I (IL-1RI) plays an important role in metastatic and invasive behaviors in pancreatic cancer, and proved that the strong expression of the α_6 _integrin subunit in pancreatic cancer tissue significantly correlated with the poor prognosis and the presence of hepatic metastases in patients with pancreatic cancer [9,10].

The plasminogen activation cascade is one critical pathway frequently implicated in cancer cell growth, invasion, and spread [10-12]. Overexpression of urokinase plasminogen activator (uPA) and uPA receptor (uPAR) have been reported in human cancer tissues, and a strong correlation has been associated between uPA and uPAR expression levels and poor prognosis and uPA is localized in primary pancreatic cancer specimens [13,14].

The activation of Ras and its downstream extracellular signal-regulated kinase/mitogen-activated protein kinase (ERK/MAPK) pathway is one of the important roles of integrin ligation [15]. Furthermore, overexpression of uPAR in cancer cells is maintained by constitutively activated ERK1-dependent signaling cascade [16]. Recently it has been demonstrated that the inhibition of the ERK/MAPK pathway suppresses the pancreatic cancer cell invasion *in vitro *[17] and colonic tumor growth *in vivo *[18]. Based on these reports, integrins in association with uPAR may activate the Ras pathway to regulate proliferative and invasive behaviors of cancer cells.

The aims of this study were to identify the role of integrins and uPA/uPAR for pancreatic cancer cell adhesive and invasive capabilities and to evaluate the correlation of uPA and integrins expression with clinicopathological characteristics of pancreatic cancer patients. We demonstrated that uPA/uPAR and α_6_β_1_-integrin play important roles in enhancement of adhesive and invasive capabilities of pancreatic cancer cells through Ras/ERK signaling pathway. Furthermore, immunohistochemical analysis demonstrated that strong expression of uPAR and α_6 _integrin was found to be independent prognostic indicator of pancreatic cancer patients. Our results suggest that IL-1α induces discernibly aggressive capability in pancreatic cancer and that these regulations can be helpful to understand biological processes for better translational treatment for pancreatic cancer patients.

## Results

### Integrins, IL-1RI, uPA and uPAR expression and alteration in pancreatic cancer cells

**Figure 1:**
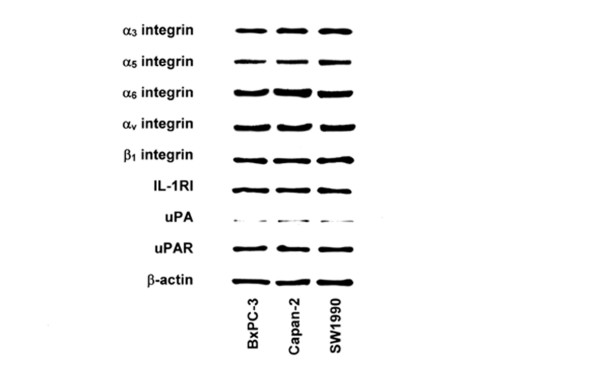
Integrins, IL-1RI, and uPAR expression in pancreatic cancer cell lines. Integrin subunits, IL-1RI, and uPAR protein expression in pancreatic cancer cell lines was determined in whole cell lysates by Western blotting analysis. Fifty micrograms of total cell lysates was separated on 10% SDS-PAGE and transferred to polyvinylidene difluoride membranes. Membranes were probed with antibodies against α_6 _integrin, α_v _integrin, β_1 _integrin, IL-1RI, and uPAR. β-actin Western blot served as a loading control.

We first analyzed three pancreatic cancer cell lines, BxPC-3, Capan-2, and SW1990, for the presence of integrin subunits, IL-1RI, uPA, and uPAR. In immunoblotting analysis, all three cell lines have expression of α_5_, α_6_, and α_v _integrin subunits. All three cell lines lacked β_4 _integrin subunits expression, while high expression of β_1 _integrin subunit was observed. The high expression of IL-1RI was observed in three cell lines. The expression of uPA and uPAR was also observed in three cell lines (Figure 1).

In flow cytometric analysis, integrin surface expression was measured since the cells were fixed and not permeabilized prior to anti-integrin antibody incubation. The expression of α_5 _and α_v _integrin subunits remained unchanged in response to rIL-1α after 24 h, while the expression of α_6 _and β_1 _integrin subunits was enhanced in all pancreatic cancer cells (Table 1). In BxPC-3 cells, the expression of α_3 _integrin subunit was enhanced in response to IL-α, while it remained unchanged in Capan-2 and SW1990 cell lines. The uPAR expression was also enhanced in response to rIL-1α after 24 h in all three cell lines. Enhancement of uPAR correlated with an increase in the cell surface expression of uPA in pancreatic cancer cells (Table 1).

**Table 1:** Alteration of integrin subunits and uPAR in pancreatic cancer cells in response to IL-1α

Antigen	IL-1α	Mean fluorescence intensity		
		
		BxPC-3	Capan-2	SW1990
α_3_integrin	-	310 ± 18	350 ± 12	411 ± 14
	+	383 ± 34^a^	344 ± 18	399 ± 16
α_5_integrin	-	40 ± 6	72 ± 9	60 ± 8
	+	44 ± 4	68 ± 8	61 ± 6
α_6_integrin	-	520 ± 21	230 ± 11	441 ± 18
	+	833 ± 44^a^	344 ± 27^a^	612 ± 32^a^
α_v _integrin	-	77 ± 8	61 ± 9	51 ± 8
	+	71 ± 7	65 ± 9	58 ± 6
β_1_integrin	-	997 ± 42	814 ± 31	810 ± 33
	+	1229 ± 41^a^	1183 ± 22^a^	1322 ± 24^a^
uPA	-	34 ± 3	42 ± 2	51 ± 5
	+	143 ± 10^a^	166 ± 13^a^	191 ± 9^a^
uPAR	-	219 ± 17	299 ± 31	291 ± 18
	+	423 ± 26^a^	588 ± 21^a^	524 ± 19^a^

IL-1α, interleukin-1α; uPA, urokinase plasminogen activator; uPAR, urokinase plasminogen activator receptor. All data are expressed as mean ± s.d. The *p*-values indicate statistical significance between data in controls and presence of IL-α. ^a ^*p *< 0.01.

### Proliferation of pancreatic cancer cells in response to IL-1α

**Figure 2:**
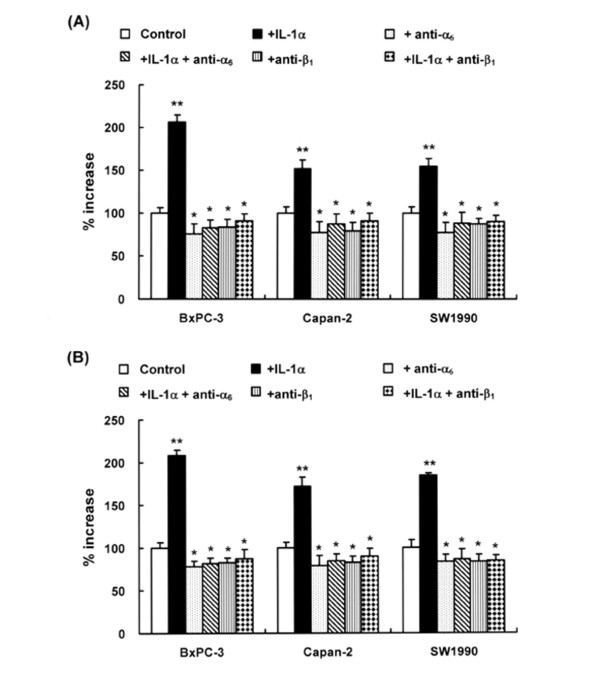
Effect of IL-1α on the proliferation of pancreatic cancer cell lines. **(A) **Cancer cell viability as a parameter of cell proliferation was assessed using the MTT assay. Three pancreatic cancer cells were treated with/without 10 ng/ml IL-1α after incubation with/without 0.5 μg/ml anti-α_6 _or anti-β_1 _integrin antibody for 24 h in serum free medium. Bars indicate the s.d. Experiments were performed in triplicate and repeated three times. **: *p *< 0.01, *: *p *< 0.05 vs. control. **(B) **The effect of IL-1α on cell growth was assessed by cell count. Three pancreatic cancer cells were treated with/without 10 ng/ml IL-1α after incubation with/without 0.5 μg/ml anti-α_6 _or anti-β_1 _integrin antibody for 24 h in serum free medium. Bars indicate the s.d. Experiments were performed in triplicate and repeated three times. **: *p *< 0.01, *: *p *< 0.05 vs. control.

We determined the proliferative response of three pancreatic cancer cell lines in response to rIL-1α for 24 h. Pancreatic cancer cells stimulated by rIL-1α showed 1.5–2.0-fold increase in proliferation compared to untreated conditions (Figure 2A and 2B). Inhibitory antibodies against α_6 _and β_1 _integrin inhibited the baseline and IL-1α-induced proliferation of all three pancreatic cancer cell lines (Figure 2A and 2B). No effect on proliferation was observed with control IgG (data not shown). These results indicate that α_6 _and β_1 _integrin subunits are involved in IL-1α-induced and normal proliferation of pancreatic cancer cells.

### Adhesion of pancreatic cancer cells in response to IL-1α

**Figure 3:**
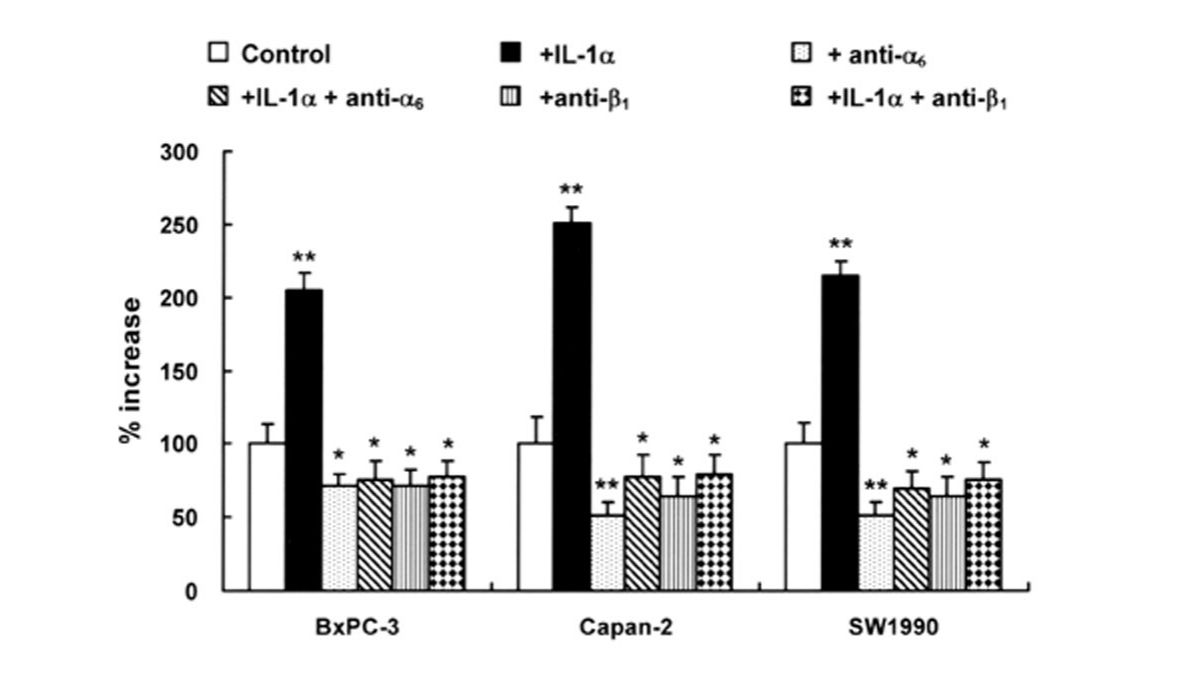
Effect of IL-1α on pancreatic cancer cell adhesion. Pancreatic cancer cells were cultured with 10 ng/ml IL-1α, with 10 ng/ml IL-1α and 0.5 μg/ml anti-α_6 _integrin antibody, or with 0.5 μg/ml anti-β_1 _integrin antibody for 24 h, after which the cell adhesion assay was performed at 37°C for 30 min. Bars indicate the s.d. **: *p *< 0.01, *: *p *< 0.05.

To determine whether IL-1α have any effect on the adhesion of pancreatic cancer cells, we investigated the adhesive response of pancreatic cancer cell lines to laminin, the putative ligand of the α_6_β_1_-integrin, in response to rIL-1α in pancreatic cancer cells. All three cell lines showed enhanced adhesion when stimulated by rIL-1α for 24 h (Figure 3). IL-1α-induced and basal adhesive response of these cell lines was suppressed by inhibitory antibodies against α_6 _and β_1 _integrin but not by control IgG.

### Migration of pancreatic cancer cells in response to IL-1α

The migration potential of pancreatic cancer cells stimulated with rIL-1α for 24 h was examined by using Matrigel-coated invasion chambers. All three pancreatic cancer cell lines showed significant enhancement of migration in the presence of IL-1α. The migration of pancreatic cancer cells correlated with an increase in cell surface bound uPA and uPAR (Table 1). IL-1α-induced migration of pancreatic cancer cells was inhibited by anti-α_6 _integrin, anti-β_1 _integrin, and anti-uPAR antibodies. Especially, each antibody inhibits the basal migration of these three cell lines (Table 2). Control IgG had no effect on the migration of pancreatic cancer cell lines. These data suggest that IL-1α-induced enhancement of α_6 _and β_1 _integrin and uPAR may have a role in enhancing the migration of pancreatic cancer cells.

**Table 2:** Migration of pancreatic cancer cells enhanced by IL-1α and its suppresion by anti-integrin, anti-uPAR antibodies

	Pancreatic cancer cells (number of migrated cells)					
	BxPC-3		Capan-2		SW1990	

Control	32.0 ± 6.24	(0)	27.7 ± 3.09	(0)	33.3 ± 3.09	(0)
IL-1α	59.2 ± 6.65 ^a^	(184.9)	47.0 ± 7.13 ^a^	(170.0)	63.2 ± 7.13 ^a^	(189.5)
IL-1α + IgG	58.3 ± 8.69 ^a^	(182.3)	45.8 ± 6.82 ^a^	(165.7)	62.5 ± 6.82 ^a^	(187.5)
anti-α_6 _integrin	21.0 ± 1.35 ^a^	(65.6)	19.9 ± 2.21 ^a^	(71.8)	23.6 ± 2.48 ^a^	(70.9)
IL-1α + anti-α_6 _integrin	23.0 ± 2.45 ^a^	(71.9)	22.3 ± 3.34 ^a^	(80.1)	23.3 ± 3.34 ^a^	(70.0)
anti-β_1 _integrin	21.1 ± 1.35 ^a^	(65.9)	23.3 ± 1.41 ^a^	(84.1)	22.1 ± 2.14 ^a^	(66.4)
IL-1α + anti-β_1 _integrin	23.7 ± 2.05 ^a^	(74.0)	22.5 ± 2.75 ^a^	(81.3)	23.7 ± 2.75 ^a^	(71.0)
anti-uPAR	25.2 ± 2.31 ^a^	(78.8)	20.1 ± 2.07 ^a^	(72.6)	24.1 ± 1.18 ^a^	(72.4)
IL-1α + anti-uPAR	24.5 ± 4.28 ^a^	(76.6)	21.2 ± 2.48 ^a^	(76.5)	24.8 ± 2.48 ^a^	(76.5)

IL-1α, interleukin-1α; uPAR, urokinase plasminogen activator receptor. All data are expressed as mean ± s.d. (% increase). The *p*-values indicate statistical significance between data in controls and presence of IL-α with/without respective antibodies. ^a ^*p *< 0.05.

### IL-1α activates Ras and downstream ERK pathway in pancreatic cancer cells

**Figure 4:**
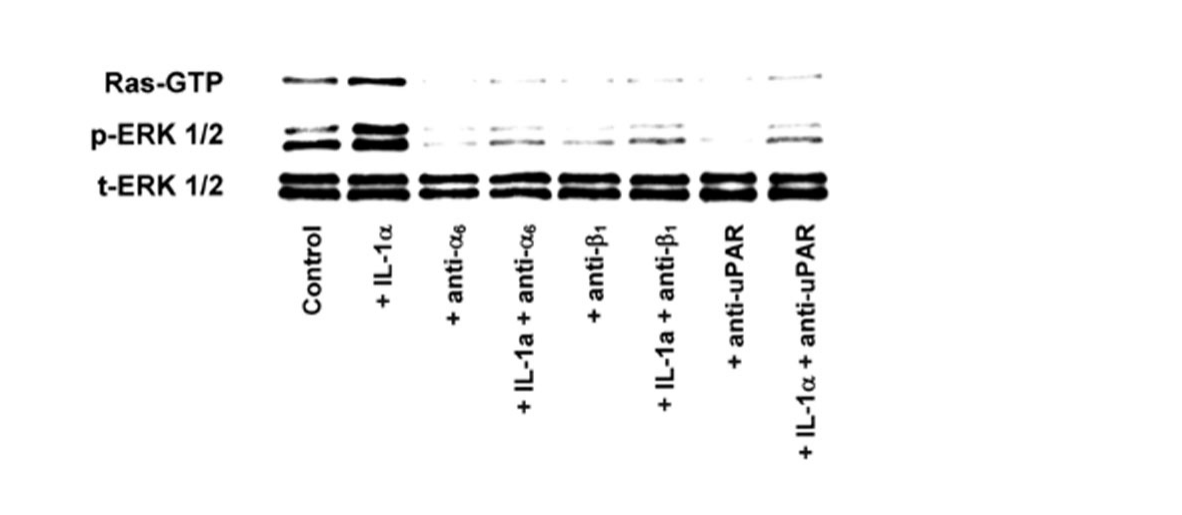
Effect of IL-1α on the activation of Ras and ERK pathway in SW1990 cell lines. Activation of Ras and downstream ERK was performed as described in Materials and Methods. SW1990 cells were serum starved for 24 h and then treated with IL-1α in the presence or absence of inhibitory antibodies for 30 min. Cell lysates were prepared according to the instructions provided in the Ras Activation Assay Kit, and affinity precipitation of GTP-bound Ras was performed using GST-tagged Raf-RBD. Levels of pull-downed Ras (Ras-GTP) were determined by anti-Ras immunoblotting. Effect of IL-1α and α_6_, β_1 _integrin and uPAR inhibitory antibodies on the baseline/IL-1α-induced activation of ERK was also examined by immunoblotting. Detection of total ERK 1/2 levels served as a loading control.

To estimate whether IL-1α can activate Ras/ERK pathway to regulate proliferation, adhesion and migration of pancreatic cancer cells, we investigated the effect of IL-1α on this pathway. IL-1α enhanced the activation of Ras, as evidenced by the increased Ras-GTP levels in pancreatic cancer cells. Activation of Ras correlated with the phosphorylation of ERK. These results indicate that IL-1α may induce activation of ERK through a Ras-dependent pathway (Figure 4). To evaluate whether α_6_β_1_-integrin and uPAR affect activation of Ras and ERK, pancreatic cancer cells were treated for 30 min with the inhibitory antibodies before being exposed to rIL-1α for 30 min. Inhibition of α_6 _and β_1 _integrin subunits and uPAR signaling pathway inhibited activation of basal/IL-1α-induced Ras and phosphorylation of ERK (Figure 4). We performed the same examinations on all three cell lines. The results of three cell lines are very similar to the presented findings, therefore, we presented the data of SW1990 cell line. These results suggest that α_6_β_1_-integrin and uPAR expression have an important role in regulating IL-1α-induced activation of signaling pathways via IL-1RI. Detection of total ERK 1/2 levels served as a loading control.

### Immunohistochemical localization of α_6 _and β_1 _integrin and uPAR in ductal adenocarcinoma of pancreas

The clinical features of 42 patients with invasive ductal adenocarcinoma of the pancreas were evaluated (Table 3). The mean age of all patients was 64.3 ± 9.1 years (range: 45 – 81 years). None had received prior chemotherapy or radiation therapy. Some patients received postoperative therapy; however, there was no difference in outcome among the various treatment modalities. The pT, pN, and pM categories were determined according to the TNM classification [19]. The M category was determined from the intraoperative findings, chest and bone radiography, ultrasonography, computed tomography, and laboratory tests reflecting bone, chest, and liver metastasis. Ten patients had synchronous or heterochronous liver metastasis. The tissue specimens were obtained at pancreatoduodenectomy (n = 35) and distal pancreatectomy (n = 7).

**Table 3:** Comparison of α_6 _integrin and uPAR expression and clinicopathological findings

		Strong co-expression both of α_6 _integrin and uPAR		^a^*p *value
		
		Yes (n = 16)	No (n = 26)	0.011
Gender	Male/Female	11/5	16/10	N.S.
Age (year)		64.4 ± 8.6	64.3 ± 9.5	N.S.
TNM stage	I/II/III/IV	2/1/6/7	7/4/12/3	N.S.
Location	H/B, T	12/4	20/6	N.S.
Liver metastasis	Yes/No	7/9	3/23	0.019
Lymph node matastasis	Yes/No	13/3	13/13	0.045
Cancer cell differentiation	P/Muc/W/Mod	1/0/4/11	2/3/13/8	N.S.
Retroperitoneal invasion	Yes/No	14/2	15/11	0.045
Intrapancreatic nerve invasion	Yes/No	14/2	17/9	N.S.
Lymphatic system invasion	Yes/No	15/1	20/6	N.S.
Venous system invasion	Yes/No	15/1	20/6	N.S.

uPAR, urokinase plasminogen activator receptor; H, head of pancreas; B, body of pancreas; T, tail of pancreas; P, papillary adenocarcinoma; Muc, mucinous carcinoma; W, well-differentiated adenocarcinoma; Mod, moderatery-differentiated adenocarcinoma. The pT, pN, and pM categories were determined according to the TNM classification [19]. The ^a^*p*-values were obtained using the Mann-Whiteny *U *test. Values of age are given mean ± s.d. N.S., no significant.

**Figure 5:**
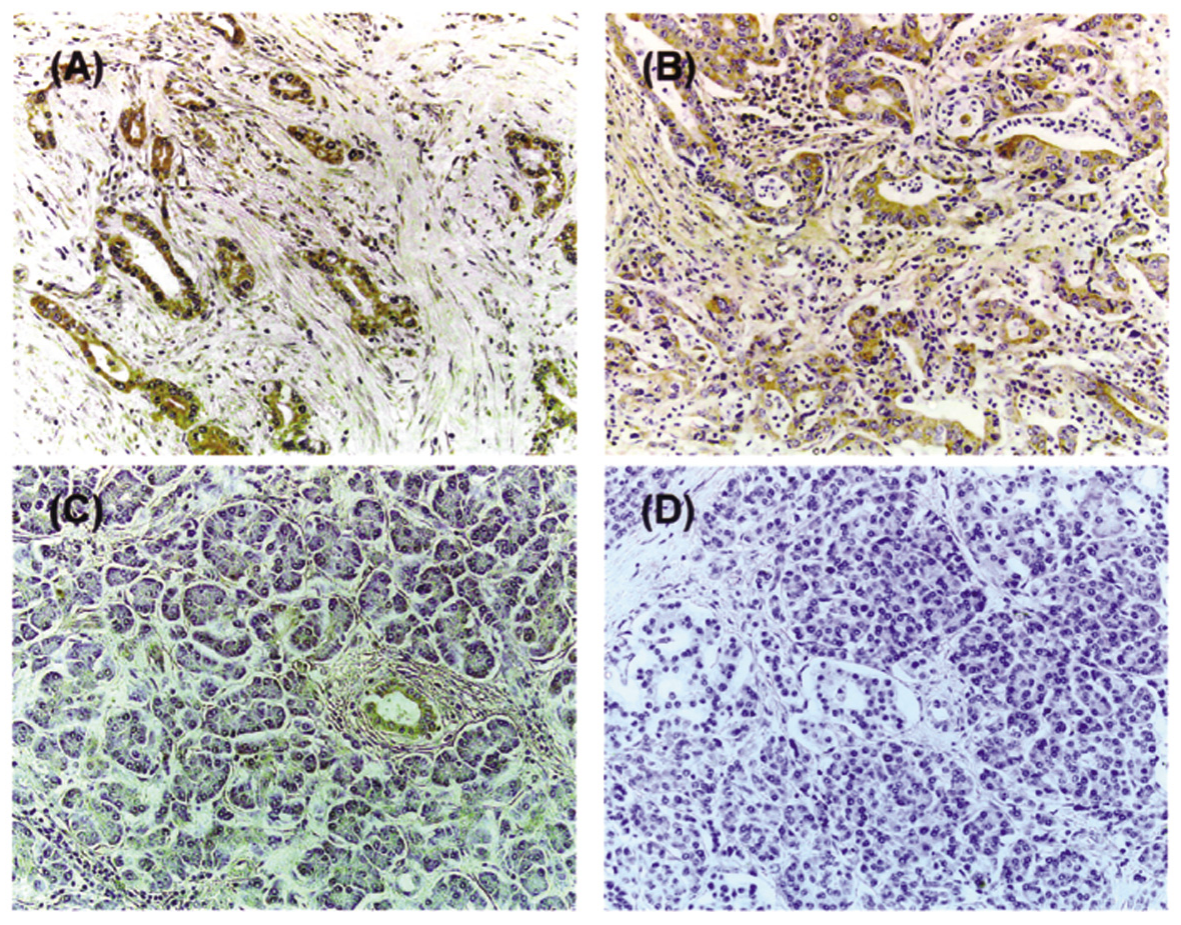
Expression of α_6 _integrin subunit and uPAR in specimens from pancreatic cancer patients. Tissue samples fixed in 10% formalin and embedded in paraffin were stained using the labeled streptavidin biotin method and specific antibodies as described in Materials and Methods. **(A) **Strong expression of the α_6 _integrin subunit in a specimen from ductal adenocarcinoma of pancreas. Magnification: ×200. **(B) **Strong expression of the uPAR in a specimen from ductal adenocarcinoma of pancreas. Magnification: ×200. **(C) **Weak expression of the α_6 _integrin subunit in a specimen from non-cancerous region of pancreas. Magnification: ×200. **(D) **The expression of uPAR was absent in a specimen from non-cancerous region of pancreas. Magnification: ×200.

Immunohistochemical expression of α_6 _and β_1 _integrin subunits and uPAR was evaluated in invasive ductal adenocarcinomas (n = 42) and duct cells in non-cancerous region of pancreas (n = 42). In 20 cancerous regions, the α_6 _integrin subunit and uPAR were strongly to moderately expressed (Figure 5A and 5B), whereas the α_6 _integrin subunit was not expressed or expressed weakly and uPAR was absent in non-cancerous region of the pancreas (Figure 5C and 5D). Weak-to-strong expression of the β_1 _integrin subunit was observed in both malignant and non-cancerous region, and there was no trend in β_1 _integrin subunit expression. A significant association was found between strong expression of α_6 _integrin subunit and uPAR in pancreatic cancer specimens (*p *= 0.011, Table 3). The α_6 _integrin subunit expression was significantly higher in malignant regions compared to non-cancerous regions of the pancreas (Table 4).

**Table 4:** Immunohistochemical evaluation of α_6 _integrin and uPAR expression in pancreatic tissues

	α_6_integrin (cases)		^a^*p*	uPAR (cases)		^a^*p*
				
	Group S	Group W		Group S	Group W	
cancerous region	20	22	< 0.01	25	17	< 0.01
non-cancerous region	5	37		0	42	

uPAR, urokinase plasminogen activator receptor. The ^a^*p*-values indicate statistical significance between cancerous region and non-cancerous region.

There was a significant association between strong co-expression of α_6 _integrin subunit with uPAR and the presence of liver metastasis (*p *= 0.019), lymph node metastasis (*p *= 0.045), and retroperitoneal invasion of pancreatic cancer (*p *= 0.045). No significant correlation was found between co-expression of α_6 _integrin subunit with uPAR and gender of patients, age, TNM stage, tumor location, cancer cell differentiation, intrapancreatic nerve invasion, lymphatic system invasion, and venous system invasion (Table 3). We also carried out a multivariate analysis of survival using the Cox's proportional hazards regression model, including each of the pathological parameters and strong expression of α_6 _integrin subunit and uPAR (Table 5). The strong expression of α_6 _integrin subunit (hazard ratio = 0.547, *p *= 0.026) and uPAR (hazard ratio = 0.491, *p *= 0.006) can be independent prognostic factors.

**Table 5:** Multiple analysis on prognosis of patients with pancreatic cancer

	Hazard Ratio	95% confidence interval	^a^*p*
Cancer cell differentiation	1.417	0.669 – 3.526	0.105
Retroperitoneal invasion	1.025	0.528 – 1.853	0.938
Intrapancreatic nerve invasion	1.604	0.605 – 1.890	0.164
Lymphatic system invasion	1.570	0.702 – 3.476	0.253
Venous system invasion	0.534	0.325 – 1.308	0.079
Lymph node metastasis	0.907	0.404 – 1.207	0.717
Strong expression of α_6 _integrin	0.547	0.295 – 0.782	0.026
Strong expression of uPAR	0.491	0.276 – 0.819	0.006

Statistical significance was indicated by ^a^*p *< 0.05.

**Figure 6:**
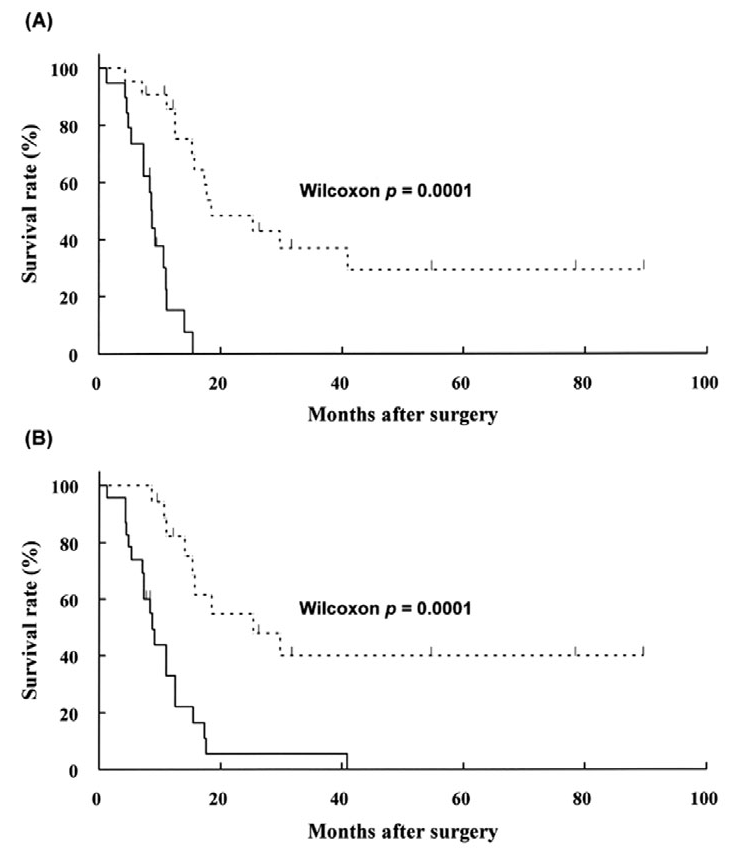
Kaplan-Meier survival curves for pancreatic cancer patients. **(A) **A comparison of survival curves between cases with (thick line) and without (broken line) strong expression of α_6 _integrin. **(B) **A comparison of survival curves between cases with (thick line) and without (broken line) strong expression of uPAR.

At the time of analysis, the median follow-up time for patients was 17.9 months (range; 1.3 – 89.6 months) after surgery. Twenty-six patients died of pancreatic cancer, and three patients died of other diseases. There was a statistically significant association between cases with and without strong expression of α_6 _integrin in poor prognosis of patients with invasive ductal adenocarcinoma of the pancreas (Figure 6A). In addition, a statistically significant association was also detected between cases with and without strong uPAR expression (Figure 6B).

## Discussion

In this study, we demonstrate that IL-1α-induced proliferation, adhesion and migration of pancreatic cancer cells correlated with activation of Ras and downstream ERK pathway. Inhibition of α_6_, β_1 _integrin, or uPAR signaling pathway inhibited IL-1α-induced activation of Ras/ERK pathway with subsequent inhibition in proliferation, adhesion and migration of pancreatic cancer cells. These observations suggest that α_6_β_1_-integrin and uPAR play a significant role in IL-1α-regulated functions of pancreatic cancer cells.

Enhancement of α_6 _integrin expression has been reported previously for cells undergoing malignant transformation such as fibroblasts [20], squamous cell carcinoma [21], hepatocytes [22], mouse epidermal keratinocytes [23], malignant melanoma [24], prostate cancer [25], and pancreatic cancer [8,9]. We previously reported that the expression of only two subunits, the α_6 _and β_1 _integrin subunits, by the high-metastatic cancer cell lines was enhanced by IL-1α, and the adhesive and invasive capability was also enhanced by IL-α[8]. In this study, we have determined the enhancement of α_6_β_1_-integrin expression by IL-1α and the subsequent increased migration of pancreatic cancer cell lines which express IL-1RI protein to Matrigel, which contains several ECM proteins. The α_6 _integrin subunit is a major laminin receptor for adhesion in laminin-rich basement membranes. In regard to the expression of α_3 _integrin which binds to collagen type I, fibronectin, and laminin with low specificity, we could not detect any changes in Capan-2 and SW1990 cell lines, whereas its expression was significantly enhanced in BxPC-3 cell lines. The enhancement of α_5 _and α_v _integrins expression was not observed in response to IL-1α in this study. Although the relative contributions of these adhesion molecules alterations appear to vary depending on the cell line and the stimulus used, in this study we can suggest that the α_6 _integrin subunit which has a strong adhesion affinity to laminin is one of the most important biological molecules for cancer cell adhesion and migration.

The strong expression of α_6 _integrin was observed in 48% of cancerous regions of the pancreas, while the α_6 _integrin subunit was weakly expressed in non-cancerous regions (*p *< 0.01). Interestingly, in non-cancerous regions of pancreatic tissues, α_6 _integrin subunit was not or only weakly expressed. The α_6 _integrin subunit is an integral part of hemidesmosomes. It is possible that the detachment of cancer cells from the pancreatic tissues and resultant metastasis formation in the target organs may be easier where α_6 _integrin subunit expression in non-cancerous regions of pancreatic tissues is weak or not observed. And the enhanced expression of the α_6 _integrin subunit via IL-1 signaling transmitted through IL-1RI may results in increased invasive and metastatic capabilities of cancer cells in cancerous tissues. In addition, the induction of microenvironment induced expression of adhesion and metastasis-related molecules may serve to regulate the process of pancreatic cancer proliferation, adhesion and invasion.

In this study, no expression of β_4 _integrin subunit was observed in three pancreatic cancer cell lines studied. The lack of β_4 _integrin subunit is consistent with the reported for prostate cancer. The progression of the cancer from intraepithelial neoplasia to invasive prostate carcinoma results in loss of β_4 _integrin expression and is replaced by alternative α_6_β_1_-integrin functions [26]. Concerning the β integrin subunit, pancreatic cancer cells that express β_1 _integrin with naturally acquired high constitutive activity were able to maintain the necessary balance of adhesion and release by means of coordinated activation and inactivation of integrin affinity [27].

In this study, we have focused in identifying some of the molecules that are regulated by IL-1α with a view to gain better understanding of the IL-1α induced molecular mechanisms that may contribute to the progression and dissemination of pancreatic cancer. We previously reported that blocking IL-1RI with neutralizing antibody inhibited the adhesion and migration of pancreatic cancer cells. We also proved that IL-1α had no demonstrable effect on pancreatic cancer cell lines without expressing IL-1RI [8]. We herein demonstrated that the proliferation of pancreatic cancer cells was enhanced by exposing to IL-1α. IL-1α also enhanced the adhesion and migration of pancreatic cancer cell lines expressing the IL-1RI, and these enhancements correlated with the enhancement of α_6_β1-integrin and uPA/uPAR expression. Based on our results, enhancement of α_6_β_1_-integrin and uPA/uPAR expression in pancreatic cancer cells occurs in the presence of IL-1RI.

The concomitant overexpression of uPA and uPAR was found to be associated with shorter survival in pancreatic cancer patients [13]. On the other hand, Harvey *et al*. reported that there were not any correlation with the co-expression of uPA and uPAR [28]. In our immunohistochemical analysis, uPAR was strongly expressed in 59.5% of cancerous regions of pancreatic cancer, whilst the expression of uPAR was absent in non-cancerous region of the pancreas. A significant association was demonstrated between strong expression of α_6 _integrin subunit and uPAR in pancreatic cancer specimens. The strong co-expression of α_6 _integrin subunit with uPAR supports our results *in vitro *and suggests that α_6_β_1_-integrin and uPAR play a significant role in aggressive functions of pancreatic cancer cells. In this study, we demonstrated a significant correlation between co-expression of α_6 _integrin subunit with uPAR and the presence of liver metastasis, lymph node metastasis, and the retroperitoneal invasion in patients with pancreatic ductal adenocarcinoma. We also found that the strong expression of α_6 _integrin subunit and uPAR correlated with the patient's poor prognosis. Furthermore, multivariate analysis demonstrated that the strong expression of α_6 _integrin subunit and uPAR can be independent prognostic indicators in patients with pancreatic ductal adenocarcinoma. These observations suggest that the diagnostic evaluation of α_6 _integrin subunit and uPAR expression might provide valuable prognostic information to aid treatment strategies for pancreatic cancer patients.

Recent reports demonstrated that integrins directly associate with uPAR to mediate cellular function [29-32]. uPAR has been reported to associate with many signaling molecules and to mediate signal transduction [33]. The α_6 _integrin/uPAR interaction has been demonstrated in human ovarian cancer cell [32] and prostate cancer cell lines [34], and these data suggest that signaling through α_6 _integrin and uPAR may be essential for ensuring cancer phenotype expression. Recently, Ahmed *et al*. reported the loss of uPA/uPAR-mediated ERK activation with downregulation of uPAR expression in colon cancer cells [35]. They also reported that the upregulation of α_6 _integrin and uPA/uPAR correlated with the activation of Ras and its downstream ERK pathway in ovarian cancer cells [32]. uPA/uPAR interaction with β_1 _integrin has been shown to activate ERK pathway [36] and disruption oft his interaction can result in loss of adhesion and tumor progression in nude mice [37]. Furthermore, it has been reported that integrin-ECM interactions activate ERK 1/2 signaling cascades [37]. We demonstrated that IL-1α stimulation and cancer cell adhesion to collagen type IV enhanced the focal adhesion kinase (FAK) protein association with β_1 _integrin and FAK phosphorylation. And these enhancements correlated with the activation of Ras/ERK signaling pathways in pancreatic cancer cells [38]. The integrin-uPAR interaction is very important as many integrin receptors activate intracellular signal pathways to fully activate cell survival and proliferation pathways [39].

## Conclusion

In summary, upregulation of α_6 _integrin subunit and uPA/uPAR correlated with the activation of Ras and downstream ERK pathways. IL-1α-induced activation of Ras and downstream ERK pathway can be inhibited by using inhibitory antibodies against α_6 _and β_1 _integrin and uPAR, consistent with the inhibition of proliferation, adhesion and migration of pancreatic cancer cells. Immunohistochemical analysis demonstrated a significant association between strong co-expression of α_6 _integrin and uPAR in pancreatic cancerous regions, and the strong expression of α_6 _integrin and uPAR was found to be independent prognostic indicators in pancreatic cancer patients. Based on these results, IL-1α induces discernibly aggressive capability in pancreatic cancer and these regulations can be helpful to understand biological processes of pancreatic cancer.

## Methods

### Cell culture

The human pancreatic cancer cell lines, BxPC-3, Capan-2, and SW1990, were from the American Type Culture Collection (Rockville, MD). The BxPC-3 cells were maintained in RPMI 1640 (Gibco BRL, Eggenstein, Germany) supplemented with 10% fetal calf serum (FCS). SW1990 and Capan-2 cells were maintained in Dulbecco modified Eagle medium (Gibco BRL) with high glucose and 10% FCS. All cells were incubated at 37°C in a humidified atmosphere of 5% CO_2 _in air.

### Tissues

Human pancreatic tissues were obtained in Department of Gastroenterological Surgery, Nagoya City University Hospital with patients' or their relatives' informed consent. Tissue samples were fixed in 10% formalin and then embedded in paraffin. Immunohistochemical studies on tumor-free pancreatic tissue were performed using non-cancerous regions of tumor-containing pancreas.

### Reagents

Recombinant human IL-1α (rIL-1α) was provided by Gibco BRL. The monoclonal antibodies (mAbs) used included anti-β_1 _(P5D2), anti-α_6 _(GoH3), anti-α_v _(AV1), and anti-β_4 _(439-9B) from Chemicon International, Inc. (Temecula, CA, USA); anti-IL-1RI (35730) from Genzyme/Techne; anti-uPA-specific antibody (#3471) and uPAR specific antibody (#3936) from American Diagnostica (Temecula, CA, USA); anti-phospho-ERK 1/2 (Thr 202/Tyr 204), anti-ERK 1 (C-16), and anti-ERK 2 (C-14) from Santa Cruz Biotechnology (Santa Cruz, CA, USA).

### Western blot analysis

The cells were lysed in lysis buffer (50 mM Tris-HCl, pH 7.5, 150 mM NaCl, 1 mM CaCl_2_, 1% Triton X-100, 0.1% SDS, 0.1% Nonidet P-40, 2 mM PMSF, 1 mM vanadate, 5 μg/ml Trasylol, 10 μM Pepstatin A and 10 μM leupeptin). Following a low-speed spin (500 rpm, 5 min) to pellet nuclei and cell debris, the supernatant fraction was further centrifuged (100,000 *g*, 30 min), and the crude plasma membranes obtained in the pellet were re-suspended in 20 mM Tris-HCl (pH 7.4). Protein concentrations were determined with a BCA protein assay kit (Pierce, Rockford, IL, USA). The amounts of samples were 50 μg per each lane. Western blot analyses were performed following SDS-PAGE. The lysates were separated by 10% SDS-polyacrylamide gel electrophoresis, transferred to polyvinylidene difluoride membranes (Immobilon PVDF; Nihon Millipore Ltd., Tokyo, Japan) and immunoblotted with each antibody.

### Flow cytometric analyses

Flow-cytometric analysis was performed using FACScan (Becton Dickinson Immunocytometry Systems, Mountain View, CA, USA). The indirect immunofluorescence method was applied to stain the cancer cells with various monoclonal antibodies as the primary antibody (stained for 30 min at room temperature), followed by the addition of the secondary antibody conjugated fluorescein isothiocyanate (Dako, Glostrup, Denmark). Results are expressed as mean fluorescence intensity for triplicate determinations.

### Cell proliferation assay

Pancreatic cancer cell proliferation was determined using the MTT [3-(4,5-dimethylthiazol-2-yl)-2,5-diphenyltetrazolium bromide dye reduction method] assay and cell count. In MTT assay, pancreatic cancer cells were seeded at a density of 2 × 10^3 ^cells/100 μl into 96-well plates and allowed to adhere overnight. Culture media were replaced, and the cells then cultured in medium alone (control) or in medium with/without 10 ng/ml of rIL-1α. After 24 h of incubation, cells were cultured for 4 h with the metabolic substrate tetrazolium salt MTT at a final concentration of 0.5 mg/ml. Formazan was detected spectorphotometically at 540 nm with a multiwell spectrophotometer (ELISA Reader; Biotek Instruments, Burlington, VT, USA).

In cell count, pancreatic cancer cells were seeded at a density of 2 × 10^5 ^cells on 35 mm well in media containing 10% FCS. After 24 h, cells were starved with 0.5% FCS for another 24 hours. Culture media was replaced to the fresh serum free media, and added rIL-1α at concentration of 10 ng/ml. After 24 h incubation, cells were washed once with phosphate-buffered saline (PBS), trypsinized, and centrifuged for 3 min at 1,500 rpm. The cell pellet was re-suspended in 2 ml of PBS and cells were counted using a light microscope.

Before the stimulating experiments with IL-1α were attempted, the lowest effective concentration was determined using rIL-1α at concentrations of 0.1 ng/ml, 0.5 ng/ml, 1.0 ng/ml, 10 ng/ml, and 100 ng/ml. A concentration of 10 ng/ml was determined to be the lowest effective concentration for stimulating experiments (data not shown). In some experiments, 0.5 μg/ml anti-α_6 _integrin or anti-β_1 _integrin mAbs was added to the cancer cells for 24 h. Before the blocking experiments were attempted, the lowest effective antibody concentration was determined using antibodies at concentrations of 0.1 μg/ml, 0.25 μg/ml, 0.5 μg/ml, 0.75 μg/ml, and 1.0 μg/ml. A concentration of 0.5 μg/ml was determined to be the lowest effective concentration for blocking experiments (data not shown). Experiments were performed in triplicate and repeated three times.

### Adhesion assay

Adhesion assay was performed as described previously with some modifications [8]. 24-well plates coated with laminin, the putative ligand of the α_6_β_1_-integrin, were obtained from Becton-Dickinson Labware (Franklin Lakes, NJ, USA). Briefly, cancer cells were incubated for 24 hours with/without rIL-1α (10 ng/ml) and then added (2 × 10^5 ^cells/well) to each well and incubated at 37°C for 30 min. The wells were then washed three times with PBS to remove unattached cells. In some experiments, 0.5 μg/ml anti-α_6 _or anti-β_1 _integrin antibodies were added to the cancer cells for >30 min prior to addition of rIL-1α.

### Migration assay

The migration response of pancreatic cancer cells in response to IL-1α was determined by using Matrigel-coated invasion chambers (Becton and Dickinson, USA). Cancer cells were added (1 × 10^5 ^cells/well) to the inner chamber of a cell culture insert and incubated at 37°C for 24 h, either with culture media containing 10 ng/ml rIL-1α or with culture media containing 10 ng/ml rIL-1α and 0.5 μg/ml anti-α_6 _integrin, anti-β_1 _integrin, or anti-uPAR antibodies. Complete medium containing 20% fetal bovine serum served as a chemo-attractant in the lower chamber. To quantitate migration, the filters were fixed in 70 % ethanol for 30 min and stained with Giemsa. Cells were removed from the upper surface of the filters by rubbing gently with a cotton-tipped applicator. Cells that had migrated through the membrane were counted in five random microscope fields of the lower filter surface.

### Ras activation assay

The activation state of Ras was determined using the Ras Activation Assay Kit provided by Upstate (Lake Placid, NY, USA). Briefly, pancreatic cancer cells were serum starved for 24 h, and then incubated in serum-free medium with/without rIL-1α (10 ng/ml) for 30 min. Cells were harvested and lysed in lysis buffer (100 mM HEPES, pH 7.5, 200 mM NaCl, 1% Nonidet P-40, 10 mM MgCl_2_, 5 mM EDTA and 10% glycerol), and supernatant prepared by centrifugation for 5 min at 4°C at 14,000 *g*. Ras-GTP from various treated lysates was "pulled down" using the GST fusion protein corresponding to human Ras binding domain of Raf-1 bound to agarose. The presence of Ras-GTP was detected by Western blotting using anti-Ras antibody (Upstate).

### Immunohistochemistry

Pancreatic tissues were studied using the labeled streptavidin biotin method [40,41]. Specimens were sectioned at 3.5-μm thick and deparaffinized. After rinsing in phosphate-buffered saline (pH 7.2), 10% bovine serum (Wako, Osaka, Japan) was applied for 10 min to block nonspecific binding. Sections were then incubated with anti-α_6 _integrin (overnight at 4°C), anti-β_1 _integrin (over night at 4°C), or anti-uPAR (60 min at 37°C) mAbs as primary antibodies. After rinsing in phosphate-buffered saline, sections were treated with biotinylated anti-mouse immunoglobulin (Ig) (Dako, Copenhagen, Denmark) for 10 min. After rinsing in phosphate-buffered saline, sections were treated with horseradish peroxidase-labeled streptavidin (Dako, Copenhagen, Denmark) for 10 minutes. The peroxidase reaction was visualized by incubating the sections with 0.02 % 3,3'-diaminobenzidine tetrahydrochloride in 0.05 M Tris buffer (pH 7.6) with 0.01 % hydrogen peroxide, followed by hematoxylin counterstaining. Negative control sections were prepared using normal mouse IgG instead of primary antibody.

### Immunohistochemical evaluation

Two observers (H.S. and H.F.) independently evaluated the immunostaining results. The concordance ratio was > 90%. Differences of opinion were resolved by reaching a consensus with the assistance of a third evaluator (Y.M.). The intensity of tissue staining was graded semiquantitatively on a 4-point scale (-, +, ++, and +++). Likewise, the proportion of cells stained was assessed on a 4-point scale (1, 0–15%; 2, 15–50%; 3, 50–85%; 4, 85–100% cells stained). To evaluate immunohistochemical findings from pancreatic cancer tissues, cases were classed in strongly staining (Group S) and weakly staining groups (Group W) by intensity and proportion of immunostaining. Immunostaining of intensity more than +++ or a staining area was more than 3 for α_6 _integrin subunit, β_1 _integrin subunit, or uPAR was defined as Group S.

### Statistical analysis

Statistical comparisons were made using the Student's *t *test for paired observations or by one-way ANOVA for multiple comparisons. The Mann-Whitney *U *test was used to compare the immunohistochemical characteristics. Differences between Kaplan-Meier survival curves based on Immunohistochemical analysis were tested with the Wilcoxon test. Multiple survival analysis was calculated according to Cox's proportional hazards model. Statistical significance was indicated by *p *< 0.05. Data are presented as mean ± standard deviations (s.d.). Each experiment was repeated three times and was carried out in triplicate.

## Abbreviations

IL, interleukin; uPA, urokinase plasminogen activator; uPAR, urokinase plasminogen activator receptor; ERK, extracellular signal-regulated kinase; ECM, extracellular matrix; IL-1RI, IL-1 receptor type I; MAPK, mitogen activated protein kinase; FAK, focal adhesion kinase; FCS, fetal calf serum; SDS-PAGE, SDS-polyacrylamide gel electrophoresis; MTT, 3-(4,5-dimethylthiazol-2-yl)-2,5-diphenyltetrazolium bromide dye reduction method; PBS, phosphate-buffered saline.

## Authors' contributions

HS carried out the Western blots, flowcytometric analysis, and the investigation of Ras activity in addition to the drafting of the manuscript. YO and HF contribute the adhesion and migration assays and statistical analyses. YM and TH performed the cell culture, adhesion assay, and the literature search. HT designed the experiments and contributed to the writing of the manuscript. TM conceived the project and aided in experimental design. All authors read and approved the final manuscript.
